# Draft Genome Sequence of Ammonia-Producing *Aeromonas* sp. MDS8 Strain MCC2167 from Sludge of a Dairy Effluent Treatment Plant

**DOI:** 10.1128/genomeA.00710-13

**Published:** 2013-09-12

**Authors:** Shaon RayChaudhuri, Amrita Saha, Thungri Ghoshal, Ashoke Ranjan Thakur

**Affiliations:** Department of Biotechnology, West Bengal University of Technology, BF-142, Sector 1, Salt Lake, Kolkata, West Bengal, Indiaa; Vice Chancellor’s Office, Techno India University, EM4/1, Sector V, Salt Lake, Kolkata, West Bengal, Indiab

## Abstract

The draft genome sequence of an amylase-, protease-, lipase-, oxidase-, and catalase-producing Gram-negative bacillus (*Aeromonas* sp. MDS8 strain MCC2167) with the ability to produce ammonia during 16 h of growth at 37°C, isolated from dairy sludge, with a size of 4,841,753 bp and a G+C content of 63.1%, is reported here.

## GENOME ANNOUNCEMENT

India is a major producer of milk, with an ever-expanding market in the dairy industry, annually producing 150,000 tons of cottage cheese, along with 2 million tons of whey carrying with it 130,000 tons of valuable nutrients (1); meanwhile, the global annual whey production is 10^8^ tons (2). The high organic content of its effluent makes the dairy industry a major source of water pollution worldwide (3), leading to serious environmental adversities. This raises a crucial question regarding the global sustenance of this industry. We present an isolate, *Aeromonas* sp. MDS8 strain MCC2167, from the sludge of a dairy effluent treatment plant with the ability to convert whey water into ammonia under unammended conditions, thus converting a waste product to a by-product. Strain MCC2167 is available at the Microbial Culture Collection at the National Centre for Cell Sciences, Pune, India.

The genome sequencing of MCC 2167, done on an Ion Torrent PGM instrument using a 316 chip, yielded a total of 1,599,675 raw reads. MIRA Assembler version 3.4.0 was used to assemble the total number of reads (1,465,596) with 46.16× coverage in 138 contigs, with the total data of 48.417 Mb sequenced with 274,598 bp as the size of the largest contig. The contigs were submitted to the Rapid Annotations using Subsystems Technology (RAST) (4) server, followed by generation of the corresponding coding sequence (CDS) information.

The important genes contained in the different contigs are as follows: genes for the ribosome hybernation protein YfiA; ferric uptake regulation protein FUR; Na^+^-driven multidrug efflux pump; Na^+^/H^+^ antiporter; transcriptional regulator LysR; hyaluronidase (GenBank no. AOTK01000001); type IV pilus biogenesis protein PilF; phosphoribosylformylglycinamidine synthase, synthetase subunit (EC 6.3.5.3)/phosphoribosylformylglycinamidine synthase, glutamine amidotransferase subunit (EC 6.3.5.3); small-subunit (SSU) ribosomal protein S1p (accession no. AOTK0100001); DNA mismatch repair protein MutS; ammonium transporter (accession no. AOTK01000007); decarboxylase family protein; DNA polymerase IV; DNA polymerase I (accession no. AOTK01000008); pantothenate:Na^+^ symporter (TC 2.A.21.1.1) (accession no. AOTK01000018); arsenic resistance protein ACR3 (accession no. AOTK01000020); nitrite reductase [NAD(P)H] large subunit; nitrite reductase [NAD(P)H] small subunit; nitrite transporter NirC (accession no. AOTK01000057); proteinase inhibitor (accession no. AOTK01000070); tRNA binding protein YgjH (accession no. AOTK01000074); putative plasmid replication protein RepB (accession no. AOTK01000104); alkaline phosphatase; NrfC protein; and NrfD protein (accession no. AOTK01000006).

### Nucleotide sequence accession numbers.

This whole-genome shotgun project has been deposited at DDBJ/EMBL/GenBank under the accession no. AOTK01000000. The version described in this paper is the first version, AOTK01000001.

## References

[1] Goyal N Gandhi DN 2009 Comparative analysis of Indian paneer and cheese whey for electrolyte whey drink. World J. Dairy Foods Sci. 4:70–72

[2] Jelen P Tossavainen O 2003 Low lactose and lactose-free milk and dairy products–prospects, technologies and applications. Aust. J. Dairy Technol. 58:161–165

[3] Vourch M Balannec B Chaufer B Dorange G 2008 Treatment of dairy industry wastewater by reverse osmosis for water reuse. Desalination 219:190–202

[4] Aziz RK Bartels D Best AA DeJongh M Disz T Edwards RA Formsma K Gerdes S Glass EM Kubal M Meyer F Olsen GJ Olson R Osterman AL Overbeek RA McNeil LK Paarmann D Paczian T Parrello B Pusch GD Reich C Stevens R Vassieva O Vonstein V Wilke A Zagnitko O 2008 The RAST server: rapid annotations using subsystems technology. BMC Genomics 9:75. 10.1186/1471-2164-9-75 18261238 PMC2265698

